# Longitudinal serum uric acid levels are not associated with dopamine transporter binding in progressive supranuclear palsy

**DOI:** 10.1007/s00702-026-03141-z

**Published:** 2026-04-06

**Authors:** Dominic Buchinger, Teodora Aleksic, Christof Brücke, Evelyn Berger-Sieczkowski, Thomas Nakuz, Tatjana Traub-Weidinger, Ivan Milenkovic

**Affiliations:** 1 Department of Neurology, Medical University of Vienna, Vienna, Austria; 2 Comprehensive Center for Clinical Neurosciences and Mental Health, Medical University of Vienna, 1090 Vienna, Austria; 3 Division of Nuclear Medicine, Department of Biomedical Imaging and Image-Guided Therapy, Medical University of Vienna, Vienna, Austria; 4 Department of Nuclear Medicine, Klinik Donaustadt, Vienna, Austria

**Keywords:** Progressive supranuclear palsy, Uric acid, Biomarker, [^123^I]FP-CIT SPECT, DAT SPECT

## Abstract

Progressive supranuclear palsy (PSP) causes rapid motor decline and severe dopaminergic dysfunction. While uric acid (UA) may act as a neuroprotective antioxidant in some neurodegenerative disorders (like Parkinson’s disease), its role in PSP remains unclear. This study evaluated the relationship between serum UA levels, measured cross-sectionally and longitudinally, and striatal dopamine transporter binding in PSP. A total of 33 PSP patients with repeated pre-[^123^I]FP-CIT SPECT UA measurements, along with 30 healthy control individuals and 30 patients with Alzheimer’s disease (AD), were retrospectively analyzed. Group and sex effects were analyzed with *t* tests and ANOVA. Effects of mean UA and longitudinal UA trajectories on FP-CIT SPECT binding in PSP were modeled using linear mixed-effects models and regressed against binding in four regions (caudate and putamen), separated into more-affected and less-affected side for both sexes. A Bayesian two-stage measurement-error model provided sensitivity analysis. UA was significantly lower in PSP (4.98 mg/dl) and AD (4.69 mg/dl) compared to healthy controls (5.71 mg/dL; *p* = 0.001). Sex had a significant effect on UA (*F*(1, 89) = 9.38, *p* = 0.003, partial η^2^ = 0.10), however, this effect was significant only in PSP (*p*< 0.001). Within PSP, UA–[^123^I]FP-CIT SPECT correlations were weak and nonsignificant, and neither UA intercept nor slope predicted [^123^I]FP-CIT SPECT binding (all *p* > 0.7). Bayesian estimates corroborated the absence of a credible relationship. In the present cohort, serum UA is reduced in PSP, primarily in females, but neither mean levels nor longitudinal changes are related to striatal [^123^I]FP-CIT SPECT binding, suggesting no clear association with dopaminergic degeneration in PSP, without precluding a potential role of uric acid at other disease stages.

**Supplementary Information:**

The online version contains supplementary material available at 10.1007/s00702-026-03141-z.

## Introduction

Progressive supranuclear palsy (PSP) is one of the most prevalent atypical Parkinsonian syndromes, characterized by early postural instability, supranuclear vertical gaze palsy, dementia and poor response to dopaminergic therapy (Litvan et al. 1997). Compared to idiopathic Parkinson’s disease (PD), PSP typically follows a more aggressive clinical course with rapid progression of motor and cognitive decline (McFarland 2016; Armstrong and McFarland 2019). These clinical differences are consistent with distinct underlying disease mechanisms, like different regional and cellular affection as well as aggregation and deposition of different proteins (Kovacs et al. 2020; Goedert et al. 2010).

Although the precise etiology of PSP is not understood, oxidative stress and neuroinflammation have been implicated as key contributors (Chirichigno et al. 2002). Uric acid (UA), the end product of purine metabolism, has attracted attention in this context due to its strong antioxidant properties, including scavenging of reactive nitrogen and oxygen species and stabilization of endogenous antioxidant systems (Squadrito et al. 2000). In PD, higher serum UA levels have been consistently associated with a reduced risk of disease onset and more favorable clinical outcomes, with several studies demonstrating correlations between serum UA levels and striatal dopamine transporter availability (Aerqin et al. 2022; Weisskopf et al. 2007; Zhang et al. 2024a; Jiang et al. 2024; de Lau et al. 2005; Chang et al. 2022; Moccia et al. 2015).

In contrast, the role of UA in PSP remains unclear. Some observational studies have suggested that higher serum UA levels may be inversely related to PSP risk (Oropesa-Ruiz et al. 2016; Schirinzi et al. 2017; Aerqin et al. 2022), whereas others have found no significant differences in serum and cerebrospinal fluid (CSF) UA levels between PSP patients and controls (Constantinescu et al. 2013; Sakuta et al. 2017; Brody et al. 2016). Nonetheless, a sex-specific reduction in serum UA levels has been observed in female PSP patients compared to male patients (Constantinescu et al. 2013). These discrepancies highlight the need for a more precise characterization of UA in PSP and its relationship with dopaminergic degeneration.

To date, no study has directly examined the association between serum UA levels and regional striatal dopamine transporter binding in PSP. In the present study, we examined this association by analyzing quantitative [^123^I]FP-CIT SPECT imaging in a substantially sized PSP cohort.

## Material and methods

### Subjects

Patients with PSP (*n* = 33) included in this study were selected retrospectively at the Movement Disorder Unit, Department of Neurology, Medical University of Vienna, Austria, between January 2015 and March 2025. All patients with PSP underwent ^123^I-FP-CIT SPECT imaging for diagnostic purposes at the Department of Biomedical Imaging and Image-Guided Therapy. PSP diagnoses were established according to the latest criteria of the Movement Disorder Society and classified as probable by an experienced movement disorder specialist (Hoglinger et al. 2017). As non-Parkinsonian controls, patients with Alzheimer’s dementia (AD, *n* = 30) were included. AD diagnoses were established according to the revised criteria for the diagnosis and staging of Alzheimer’s disease proposed by the Alzheimer’s Association Workgroup (Jack et al. 2024). Healthy controls (HC, *n* = 30) were retrospectively identified from general outpatient clinics among individuals presenting with non-neurological conditions (e.g., routine check-ups or minor somatic complaints). All included participants were systematically reviewed for medical comorbidities and medications known to influence serum uric acid levels (e.g., renal dysfunction, thyroid disease, malignancy, alcoholism, gout, or treatment with urate-lowering medication), and none were identified in any group.

For all included PSP patients, the last documented motor status and clinical information prior to ^123^I-FP-CIT SPECT imaging were reviewed to categorize disease severity. Patients were classified as having mild disease when they were ambulatory without major postural instability (including PSP with oculomotor variant, PSP with Parkinsonism or early/mild PSP with Richardson syndrome) and as having severe disease when marked postural instability, frequent falls, or the need for walking aids or wheelchair use was documented (PSP with corticobasal syndrome, late PSP with Richardson syndrome). All available serum UA levels prior to ^123^I-FP-CIT SPECT imaging were collected retrospectively from our internal patient data system and the available archived hospital database. We accepted values up to 30 days prior to ^123^I-FP-CIT as associated with the investigation. None of the included PSP patients were receiving medications known to significantly affect dopamine transporter binding (e.g., bupropion, psychostimulants, or selective serotonin/norepinephrine reuptake inhibitors) at the time of ^123^I-FP-CIT SPECT imaging, as verified through review of the available medical records and patient-reported documentation prior to ^123^I-FP-CIT SPECT (Chahid et al. 2023). The study was approved by the local ethics committee (EK Nr. 1265/2020).

### ^123^I-FP-CIT SPECT image acquisition, processing and image analysis

Three to 4 h prior to the imaging start ~ 185 MBq ^123^I-FP-CIT was injected intravenously (specific activity > 185 MBq/nmol; radiochemical purity > 99%; produced as DaTSCAN according to GMP criteria at GE Healthcare, Eindhoven, The Netherlands). For SPECT imaging, a three-head gamma camera (IRIX 465, Philips Medical Systems, MN Divion) equipped with a fan beam, low-energy, and high-resolution collimators was used. Acquisition parameters, including the step-and-shoot mode for over 30 min, were used. Over 360° 60 projection angles were taken using a 128 × 128 matrix. Reconstruction was performed using filtered projections using a Shepp and Logan filter. The triple-energy window method for scatter compensation and a uniform Chang attenuation correction were applied to compensate for photon attenuation using a uniform attenuation coefficient of 0.15/cm. The reconstructed I-FP-CIT images were subsequently reoriented and normalized automatically using BRASSTM (HERMES Medical Solutions, Stockholm, Sweden). Following automatic alignment, all scans were inspected visually and manually realigned to fit the predefined template where necessary. The template volumes of interest (VOIs) were predefined using BRASS software, Version 4 (Booij et al. 2001; Oh et al. 2012). For count concentration estimation in striatal (S) and background occipital (O) VOIs. Using these activity concentrations in VOIs, striatal uptake ratios defined as (S–O)/O of specific tracer binding were calculated. Similar procedures were used to split the striata in the caudates and putamina. We defined the clinically more severely affected side based on the measured data and clinical observations. As PSP typically affects both hemispheres in a largely symmetric manner, the striatal side with lower ^123^I-FP-CIT SPECT binding was considered to be the more severely affected side.

### Statistics

All analyses were conducted using R (version 4.3.2; R Foundation for Statistical Computing, Vienna, Austria) within the RStudio environment (version 2023.09; Posit Software, Boston, MA). Data distributions were assessed using histograms and density plots. Categorical variables were summarized as frequencies and percentages, and continuous variables were reported as means with 95% confidence intervals or medians with interquartile ranges (IQR). The assumptions of normality and homogeneity of variance were verified using the Shapiro–Wilk and Levene’s tests, respectively. Bonferroni correction was applied to adjust for multiple comparisons.

Differences in serum UA levels between males and females within each diagnostic group were tested using Welch's independent-samples *t*-test, one- or two-way analysis of variance (ANOVA) with group and sex as fixed factors, including the interaction term. For this analysis, individual mean values were calculated within subjects using the arithmetic means of all valid observations. Pairwise comparisons of the estimated marginal means were performed using the *emmeans* package (Tukey adjustment for multiple testing). To complement significance testing, effect sizes were calculated and reported as partial eta squared (η^2^), reflecting the proportion of variance attributable to each factor. Associations between mean UA levels and striatal dopamine transporter availability in the more-affected and less-affected caudate and putamen regions, as measured by ^123^I-FP-CIT SPECT were examined using Pearson’s correlations, interpreted as weak (*r* < 0.3), moderate (0.3 ≤ *r* ≤ 0.5), or strong (*r* > 0.5).

We adopted a two-stage modeling framework to first estimate individual longitudinal UA trajectories (Stage 1) and then relate these subject-specific parameters to striatal dopamine transporter availability measured by ^123^I-FP-CIT SPECT (Stage 2), thereby accounting for both within-subject variability and measurement errors in the predictors.

#### UA trajectory modelling (stage 1)

We first characterized each participant’s longitudinal UA profile by fitting a linear mixed-effects model. Longitudinal UA profiles were modelled using linear mixed-effects models with random intercepts and slopes (*lme4*). For each participant, best linear unbiased predictors of the UA intercept and slope, and the standard error of the slope, were extracted (*arm* package). Sex differences in slopes were tested within diagnostic groups, and an additional linear model including a sex × group interaction was fitted.

#### Endpoint regression (stage 2)

To relate UA trajectories to striatal dopamine transporter availability, ^123^I-FP-CIT SPECT–derived SUVs in the four striatal regions (more affected/less affected caudate and putamen) were regressed on individual UA intercept and slope estimates. Models were weighted by the inverse variance of the UA slope, so that participants with more precisely estimated slopes contributed more strongly to the analysis.

To validate our simple two-stage- regression, we implemented a Bayesian two-stage measurement error model in *brms* as a sensitivity analysis. To this end, we implemented a Bayesian linear mixed-effects model using the *brms* package in R (Bürkner 2017). Each participant’s standardized UA slope (derived from the Stage 1 longitudinal model) was entered as a predictor of regional DAT values (caudate more affected, caudate less affected, putamen more affected, and putamen less affected). Sex and region were included as fixed effects, and subject-specific intercepts were modeled as random effects to account for repeated measures across regions. All predictors were standardized before the analysis. The model used weakly informative priors (normal[0, 0.5] for fixed effects, Student-t[3, 0, 2.5] for the intercept, and exponential [1] for variance parameters) and was estimated using four Markov chains (4,000 iterations per chain; 1,000 warm-up). Convergence was assessed using the potential scale reduction statistic (R̂ < 1.01) and effective sample size diagnostics. Posterior summaries are reported as medians with 95% credible intervals (CrI). Practical equivalence was evaluated using a region of practical equivalence (ROPE) of ± 0.1 SD and the probability of direction (pd) index, as implemented in the *bayestestR* package.

## Results

After excluding PSP patients with insufficient UA values (at least three documented values prior to ^123^I-FP-CIT SPECT), 33 patients diagnosed with progressive supranuclear palsy (PSP) were identified, with the mean disease duration at the time of DAT imaging of 39 months (SD 25). Further, 30 healthy controls and 30 patients with Alzheimer’s disease (AD) with at least 3 UA values (without ^123^I-FP-CIT SPECT) were selected. The demographic characteristics of the cohort are shown in Table 1.

**Table 1:** The demographic characteristics and mean ^123^I-FP-CIT SPECT values per region

Group	Total (*n*)	Mean age (y)	Median age (y)	Caudate more affected	Caudate less affected	Putamen more affected	Putamen less affected	Male (%)	Female (%)
Control AD	30 30	69.1 70.9	70.7 72.6	– –	– –	– –	– –	18 (60.0%) 14 (46.7%)	12 (40.0%) 16 (53.3%)
PSP	33	67.0	66.6	1.34 ± 0.65	1.63 ± 0.71	0.93 ± 0.57	1.24 ± 0.69	18 (54.5%)	15 (45.5%)

Values are presented as mean ± standard deviation unless otherwise stated. More affected and less affected sides denote the striatal hemispheres with lower and higher dopamine transporter binding, respectively. Striatal dopamine transporter values were not available in healthy controls and are therefore not shown (–). Abbreviations: *PSP* progressive supranuclear palsy, *SPECT* single photon emission computed tomography, *DAT* dopamine transporter, ^123^I-FP-CIT ioflupane iodine-123

**Fig. 1:**
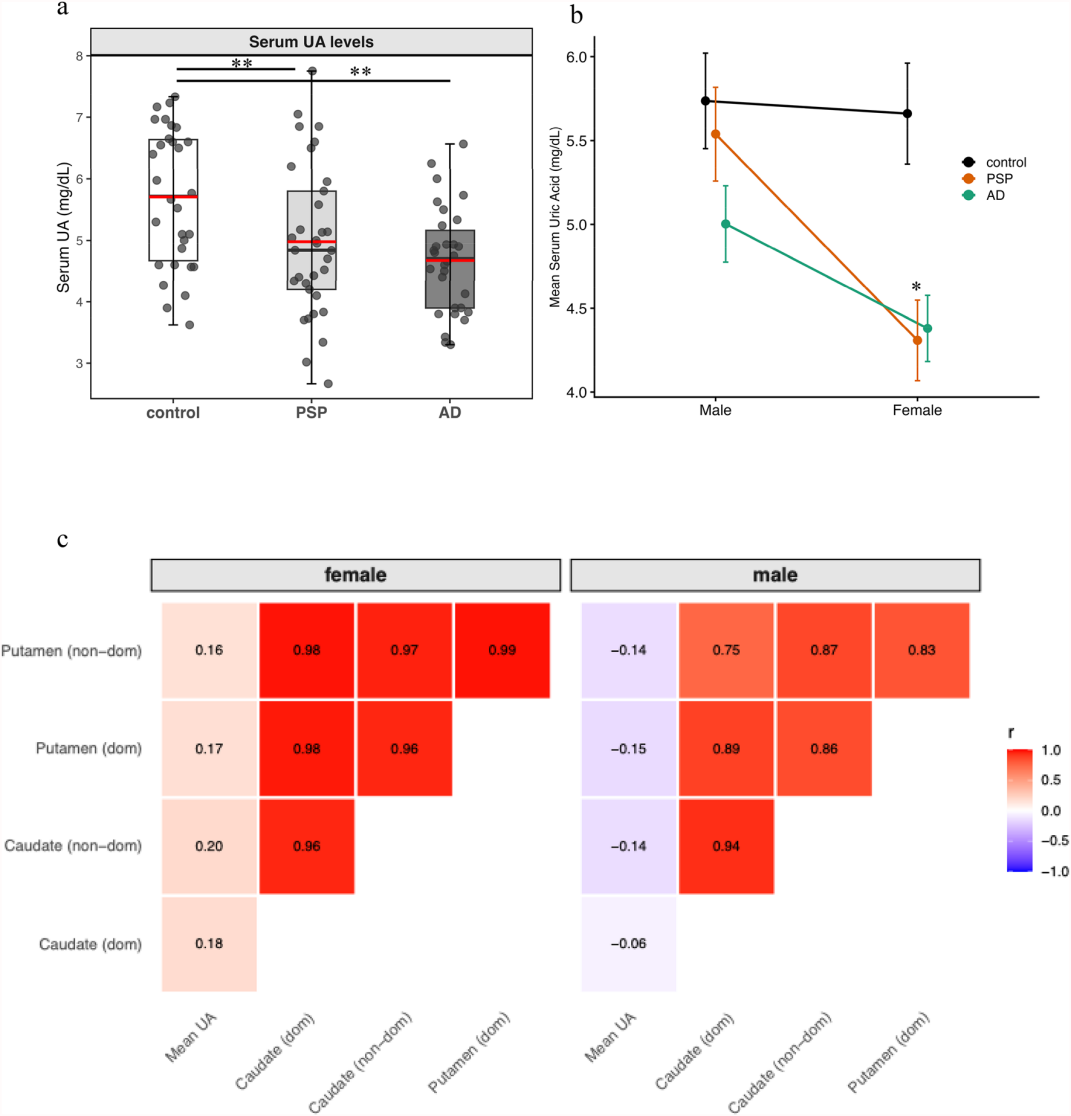
**a** Boxplots show the distribution of mean serum UA concentrations (mg/dL) between groups. Black horizontal lines inside the boxes indicate the group median, the boxes represent the interquartile range (IQR), and the whiskers show the data range, excluding outliers. The red horizontal lines denote the group means. **p < 0.002. **b** Sex-specific differences in serum uric acid levels between PSP, AD, and healthy controls. Mean serum UA levels (± SE) stratified by diagnostic group and sex. **c** Correlation matrix of serum UA and striatal DAT binding in PSP patients by sex. Pairwise Pearson correlations between mean serum UA levels and striatal DAT binding ratios in four brain regions, stratified by sex. Only the lower triangle is shown to avoid redundancy. In female patients, serum UA showed weak positive correlations with all striatal regions (*r* = 0.16–0.20), while striatal regions were highly intercorrelated (*r* = 0.96–0.99). In male patients, serum UA showed weak negative correlations with striatal regions (*r* = − 0.06 to – 0.14), with moderate to strong intercorrelations among brain regions (*r* = 0.75–0.94). Color intensity represents correlation strength (blue = negative, red = positive)

Serum UA differed significantly across diagnostic groups (*F*(2, 90) = 7.17, *p* = 0.001, η^2^ = 0.14), indicating that diagnosis accounted for 14% of the variance in UA levels (Fig. 1a). Post hoc Tukey comparisons showed that controls had higher UA levels compared to PSP (*p* = 0.027) and AD patients (*p* = 0.002), whereas PSP and AD did not differ (*p* = 0.596). Within the PSP group, no significant difference in UA levels was observed between mild and severe phenotypes (mild: M = 4.91 mg/dL; severe: M = 5.06 mg/dL; Welch’s t(24.22) =  − 0.33, p = 0.74).

To account for potential sex effects, a two-way ANOVA including diagnosis and sex was performed (Fig. 1b). Diagnosis remained a significant predictor of UA levels (*F*(2, 89) = 7.83, *p* < 0.001, partial η^2^ = 0.15), and sex also significantly influenced UA (*F*(1, 89) = 9.38, *p* = 0.003, partial η^2^ = 0.10). The diagnosis × sex interaction was not significant (*p* = 0.093). Nevertheless, exploratory simple-effects contrasts suggested a significant sex difference within PSP (male–female difference = 1.23 mg/dL, *p* = 0.0009), whereas sex differences were not significant within controls (*p* = 0.845) or AD (*p* = 0.101) (Fig. 1b, Supplementary Fig. 1a).

Next, we investigated whether the mean patients’ serum UA levels were associated with FP-CIT SPECT binding to striatal dopamine transporters. The mean longitudinal follow-up period for available serum UA measurements was 8.6 years (SD 6.8). Within the PSP group, correlations between mean serum UA levels and FP-CIT SPECT–derived striatal dopamine transporter availability were examined separately for males and females across the four regions for each patient. In female patients with PSP, we found weak positive correlations with ^123^I-FP-CIT SPECT –derived SUV (r = 0.16–0.20, all p > 0.48, Fig. 1C), whereas in male patients with PSP, correlations were weakly negative (r =  − 0.06 to − 0.15, all p > 0.54). None of these associations reached statistical significance, and Fisher’s r-to-z tests indicated no significant sex differences (all p > 0.37). Overall, these findings demonstrate that within the PSP group, serum UA levels were not significantly associated with striatal SUV in either sex (Fig. 1C). Although the mixed-effects model did not yield formally significant interactions, visual inspection of the fitted slopes suggested a possible sex-specific trend in the relationship between mean patients’ serum UA and ^123^I-FP-CIT SPECT –derived SUV in severe PSP cases. Moreover, in mild PSP, the association between UA and SUV was similar for men and women. In severe PSP, however, female patients tended to show a positive association between UA and SUV, whereas male patients showed a negative trend (Supplementary Fig. 1b).

To further explore whether longitudinal changes (rather than single values) may affect the SUV, we investigated the longitudinal relationship between serum UA and SUV using a two-step random coefficient growth curve model. To this end, we regressed regional SUV values on each participant’s UA intercept and slope (derived from individual UA time-series models) while controlling for sex, disease duration and number of collected values. Across all four striatal regions (caudate and putamen, more affected and less affected sides), neither UA intercept nor slope significantly predicted SUV (all *p* > 0.7). The effects of sex were also not significant. The model fit indices indicated a minimal explained variance (adjusted R^2^ ≈ 0).

To validate these findings and account for estimation uncertainty in Stage 1 modelling, we employed a Bayesian, two-stage measurement error model. The model converged well (all R̂ = 1.00, ESS > 4000). The posterior median estimate for the standardized UA slope effect was − 0.03 (95% CrI [− 0.41, 0.35]), and for the UA slope × sex interaction, − 0.02 (95% CrI [− 0.71, 0.68]). The main effect of sex was 0.01 (95% CrI [− 0.54, 0.57]). Region effects showed higher ^123^I-FP-CIT SPECT binding values in the caudate than in the putamen (caudate_less_affected: β = 0.26 [0.21, 0.31]; putamen_more_affected: β =  − 0.33 [− 0.38, − 0.29]; putamen_less_affected: β =  − 0.04 [− 0.09, 0.01]). The residual SD of the model was 0.22 (95% CrI [0.21, 0.23]). The 95% credible intervals for both UA-related predictors overlapped with zero, and the slope effect, in particular, was characterized by substantial uncertainty in prediction. Crucially, the results of the measurement error–corrected model were fully concordant with the initial two-stage analysis: the diagnostic group (PSP) remained the only reliable predictor of striatal dopaminergic degeneration, whereas the UA intercept and slope effects were negligible and non-significant. This consistency reinforces the robustness of our findings regarding the first-stage estimation variability (Supplementary Table 1).

## Discussion

In this study, we investigated the relationship between serum uric acid (UA) levels and striatal ^123^I-FP-CIT SPECT–derived SUV across a cohort of patients with progressive supranuclear palsy (PSP). To our knowledge, this is the first study to integrate repeated UA sampling with dopaminergic imaging in a substantial PSP cohort. Our findings provide new insights into sex- and disease-specific interactions between UA and striatal dopamine transporter availability in patients with PSP. Firstly, we noted that mean serum UA levels were significantly lower in PSP than in healthy controls. This effect was driven almost entirely by female patients, who showed markedly reduced UA levels relative to male patients with PSP and female controls. Secondly, serum UA levels were not significantly associated with FP-CIT SPECT–derived striatal dopamine transporter availability in patients with PSP. Finally, longitudinal UA changes were not related to striatal dopamine transporter (DAT) availability.

Multiple meta-analyses and cohort studies have shown that serum uric acid levels are significantly lower in PD patients compared to healthy controls (Wen et al. 2017; Shen and Ji 2013; Annanmaki et al. 2007). Many of those studies associated lower UA levels with increased PD risk, faster disease progression, and greater severity, including both motor and non-motor symptoms (Wen et al. 2017; Danau et al. 2022; Koros et al. 2023; van Wamelen et al. 2020). Interestingly, in PD this reduction was evident in both sexes, with men showing a slightly greater reduction (− 0.66 vs. − 0.53 compared to controls) (Wen et al. 2017; Shen and Ji 2013). This contrasts our observations, in which female patients with PSP showed significantly lower UA levels than both controls and male patients with PSP. Given the non-significant sex-specific changes of UA in controls and AD, this pattern may suggest that sex-related biological factors differentially influence UA regulation across neurodegenerative disorders, with a potentially heightened relevance in PSP. Notably, although males with AD demonstrated numerically higher UA levels than females (mean difference ≈ 0.6 mg/dL, compared to ≈ 1.2 mg/dL in PSP), this difference did not reach statistical significance in AD. Thus, while a sex effect in AD cannot be excluded, it appeared less pronounced than in PSP. Overall, UA levels were significantly lower in AD then in PSP, indicating disease-specific patterns. Estrogen is known to promote uricosuria and is associated with lower UA levels in women, and sex-dependent variation in renal urate transporters such as SLC2A9 and ABCG2 further contributes to this dimorphism (Hak and Choi 2008; Brandstatter et al. 2008; Merriman 2015). Potential differences between PD and PSP, and AD may reflect distinct underlying pathogenetic mechanisms, including mainly presynaptic vs. postsynaptic dopaminergic degeneration and divergent proteinopathies (alpha-synucleinopathy vs. 4R-tauopathy vs. mixed 3R/4R-tauopathy with β-amyloid accumulation) (Kovacs 2019; Goedert et al. 2010). However, further studies are needed to clarify the mechanistic basis of these observations, particularly across broader groups of Parkinsonian syndromes.

In our analysis neither mean UA levels nor their temporal fluctuations (i.e., trajectories of change) exhibited any discernible association with striatal DAT availability in patients with PSP. Likewise, neither baseline UA nor its rate of change predicted FP-CIT SPECT–derived DAT measures in any striatal region. The models explained virtually no variance in DAT availability. This result was corroborated by the Bayesian measurement-error–corrected analysis, despite being more robust compared to frequentist statistics. Within the PSP cohort, UA levels did not differ between mild and severe phenotypes. Although these comparisons did not reach statistical significance, exploratory analyses in female patients hinted at stage-dependent divergence, with trends toward inverse associations in milder disease and positive relationships in more advanced stages.

In contrast, prior reports in PD have suggested that lower serum UA levels correlate with reduced striatal DAT availability (Baik et al. 2020) potentially reflecting more severe dopaminergic neuron degeneration (Kovacs et al. 2008). This association is observed across PD motor subtypes, with the lowest UA and DAT levels in patients with postural instability and gait disorder compared to tremor-dominant subtypes (Wen et al. 2017; Moccia et al. 2015). Further, some studies provided evidence for the association between UA and disease progression or severity, potentially with a stronger effect in men (Shen and Ji 2013; Lee et al. 2020, 2018). However, the absence of any association in PSP in our study suggests that peripheral UA dynamics may be insufficiently sensitive to central dopaminergic degeneration in this disorder. This interpretation aligns with findings from Hasimoglu et al., who likewise reported lack of relationship between longitudinal UA changes and dopaminergic or clinical progression in PD (Hasimoglu et al. 2020), and is further compatible with emerging genetic and longitudinal evidence suggesting that urate may function more as a peripheral correlate than as a mechanistically relevant neuroprotectant (Wang and Tang 2024; Fazlollahi et al. 2022; Zhang et al. 2024b). It should be borne in mind that the present study assessed serum UA levels exclusively, which may not accurately reflect CSF or CNS concentrations and thus may be of limited utility as a biomarker of central neurodegeneration. Peripheral UA alterations may instead be governed primarily by systemic and sex-dependent factors that transcend diagnostic categories, rather than indexing disease-specific neurobiological processes.

Evidence from other neurodegenerative conditions suggests stage-dependent variation in uric acid levels, as well as potential discrepancies between serum and CSF measurements (Maetzler et al. 2011). In Parkinson’s disease, elevated uric acid has been linked to earlier disease phases and, in certain cohorts, more favorable outcomes, whereas lower concentrations tend to predominate in advanced stages. Such patterns have been interpreted as indicative of a shift from antioxidant buffering toward oxidative stress–driven or secondary reactive mechanisms as neurodegeneration progresses (Ikeda et al. 2011). Nonetheless, these observations remain heterogeneous and underscore the need for longitudinal studies assessing uric acid dynamics in both serum and CSF.

Several limitations should be considered when interpreting our findings. First, we were unable to account for several confounding factors that may influence serum uric acid levels, such as dietary assessment, including daily alcohol consumption and BMI, which could have introduced some residual variability. Second, PSP diagnoses were based on established clinical criteria and confirmed by experienced movement disorder specialists but not neuropathologically confirmed. Third, as this was a single-center study using a specific ^123^I-FP-CIT SPECT acquisition and analysis protocol, the generalizability of our imaging findings to other centers may be limited, despite the advantage of reduced methodological variability. Fourth, although clinically substantial, statistically rather moderate sample size within PSP subgroups—especially in sex-stratified analyses—may have limited our ability to detect subtle associations or interaction effects. Lastly, due to the retrospective design, standardized clinical severity scales such as the PSP Rating Scale were not systematically available at the time of serum uric acid sampling and [^123^I]FP-CIT SPECT acquisition, limiting correlations with clinical disease severity.

In conclusion, our findings suggest that peripheral uric acid levels may provide only limited insight into central dopaminergic degeneration in PSP. Although UA was reduced—particularly in female patients—neither average concentrations nor longitudinal changes showed measurable associations with striatal DAT availability. Together with prior PD-based evidence, Mendelian randomization analyses and the absence of disease-specific effects across additional neurological comparison groups included in this study, these results may indicate that urate might rather reflect peripheral physiological processes than disease-specific neurodegeneration. While these observations do not rule out context-dependent, disease-stage related or sex-modulated roles of UA, they further highlight the need for multimodal biomarker approaches and for further investigation into the biological mechanisms underlying UA-related sex differences in PSP in larger cohorts.

## Supplementary Information

Below is the link to the electronic supplementary material.

Supplementary Material 1 Supplementary Fig. 1. (a) Mean serum UA levels stratified by sex and diagnostic group. Bars represent group means, and error bars indicate standard error of the mean (SEM). Darker bars represent males, lighter bars represent females. (b) Associations between serum uric acid and striatal dopamine transporter binding in PSP patients stratified by disease severity and sex. Scatter plots showing the relationship between serum UA levels (x-axis) and striatal DAT binding ratios (y-axis, measured as standardized uptake value ratio, SUVR) across four brain regions. Data are stratified by disease severity (Mild: top row; Severe: bottom row) and sex (Female: orange circles; Male: green triangles). Each panel represents a different striatal region: Caudate (more_affected), Caudate (less_affected), Putamen (more_affected), and Putamen (less_affected). Linear regression lines with 95% confidence intervals (shaded areas) are shown for each sex. In mild disease, female patients generally showed positive associations between UA and DAT binding (particularly in caudate regions), while male patients showed relatively flat or negative trends. In severe disease, both sexes showed predominantly flat or negative associations across all regions, suggesting that the neuroprotective association of UA may be disease stage-dependent. None of the associations reached statistical significance

Supplementary Material 2

## Data Availability

The datasets generated and analyzed during the current study are available from the corresponding author on reasonable request due to ethical restrictions and data protection regulations.

## References

[CR1] Aerqin Q, Jia SS, Shen XN, Li Q, Chen KL, Ou YN, Huang YY, Dong Q, Chen SF, Yu JT (2022) Serum uric acid levels in neurodegenerative disorders: a cross-sectional study. J Alzheimers Dis 90(2):761–773. 10.3233/JAD-220432 36189590 10.3233/JAD-220432

[CR2] Annanmaki T, Muuronen A, Murros K (2007) Low plasma uric acid level in Parkinson’s disease. Mov Disord 22(8):1133–1137. 10.1002/mds.21502 17443703 10.1002/mds.21502

[CR3] Armstrong MJ, McFarland N (2019) Recognizing and treating atypical Parkinson disorders. Handb Clin Neurol 167:301–320. 10.1016/B978-0-12-804766-8.00016-9 31753139 10.1016/B978-0-12-804766-8.00016-9

[CR4] Baik K, Chung SJ, Yoo HS, Lee YH, Jung JH, Sohn YH, Lee PH (2020) Sex-dependent association of urate on the patterns of striatal dopamine depletion in Parkinson’s disease. Eur J Neurol 27(5):773–778. 10.1111/ene.14152 31994785 10.1111/ene.14152

[CR5] Booij J, Speelman JD, Horstink MW, Wolters EC (2001) The clinical benefit of imaging striatal dopamine transporters with [123I]FP-CIT SPET in differentiating patients with presynaptic parkinsonism from those with other forms of parkinsonism. Eur J Nucl Med 28(3):266–272. 10.1007/s002590000460 11315592 10.1007/s002590000460

[CR6] Brandstatter A, Kiechl S, Kollerits B, Hunt SC, Heid IM, Coassin S, Willeit J, Adams TD, Illig T, Hopkins PN, Kronenberg F (2008) Sex-specific association of the putative fructose transporter SLC2A9 variants with uric acid levels is modified by BMI. Diabetes Care 31(8):1662–1667. 10.2337/dc08-0349 18487473 10.2337/dc08-0349 PMC2494626

[CR7] Brody DM, Litvan I, Warner S, Riley DE, Hall DA, Kluger BM, Shprecher DR, Cunningham CR (2016) Relationship between uric acid levels and progressive supranuclear palsy. Mov Disord 31(5):663–667. 10.1002/mds.26535 26890571 10.1002/mds.26535 PMC5258200

[CR8] Bürkner P-C (2017) brms: an R package for Bayesian multilevel models using stan. J Stat Softw 80(1). 10.18637/jss.v080.i01

[CR9] Chahid Y, Sheikh ZH, Mitropoulos M, Booij J (2023) A systematic review of the potential effects of medications and drugs of abuse on dopamine transporter imaging using [(123)I]I-FP-CIT SPECT in routine practice. Eur J Nucl Med Mol Imaging 50(7):1974–1987. 10.1007/s00259-023-06171-x 36847827 10.1007/s00259-023-06171-x PMC10199883

[CR10] Chang H, Wang B, Shi Y, Zhu R (2022) Dose-response meta-analysis on urate, gout, and the risk for Parkinson’s disease. NPJ Parkinsons Dis 8(1):160. 10.1038/s41531-022-00433-5 36418349 10.1038/s41531-022-00433-5 PMC9684547

[CR11] Chirichigno JW, Manfredi G, Beal MF, Albers DS (2002) Stress-induced mitochondrial depolarization and oxidative damage in PSP cybrids. Brain Res 951(1):31–35. 10.1016/s0006-8993(02)03101-3 12231453 10.1016/s0006-8993(02)03101-3

[CR12] Constantinescu R, Andreasson U, Holmberg B, Zetterberg H (2013) Serum and cerebrospinal fluid urate levels in synucleinopathies versus tauopathies. Acta Neurol Scand 127(2):e8-12. 10.1111/ane.12012 22998191 10.1111/ane.12012

[CR13] Danau A, Dumitrescu L, Lefter A, Popescu BO (2022) Serum uric acid levels in Parkinson’s Disease: a cross-sectional electronic medical record database study from a tertiary referral centre in Romania. Medicina (Kaunas). 10.3390/medicina58020245 35208569 10.3390/medicina58020245 PMC8877142

[CR14] de Lau LM, Koudstaal PJ, Hofman A, Breteler MM (2005) Serum uric acid levels and the risk of Parkinson disease. Ann Neurol 58(5):797–800. 10.1002/ana.20663 16240356 10.1002/ana.20663

[CR15] Fazlollahi A, Zahmatyar M, Alizadeh H, Noori M, Jafari N, Nejadghaderi SA, Sullman MJM, Gharagozli K, Kolahi AA, Safiri S (2022) Association between gout and the development of Parkinson’s disease: a systematic review and meta-analysis. BMC Neurol 22(1):383. 10.1186/s12883-022-02874-0 36221048 10.1186/s12883-022-02874-0 PMC9552480

[CR16] Goedert M, Clavaguera F, Tolnay M (2010) The propagation of prion-like protein inclusions in neurodegenerative diseases. Trends Neurosci 33(7):317–325. 10.1016/j.tins.2010.04.003 20493564 10.1016/j.tins.2010.04.003

[CR17] Hak AE, Choi HK (2008) Menopause, postmenopausal hormone use and serum uric acid levels in US women–the Third National Health and Nutrition Examination Survey. Arthritis Res Ther 10(5):R116. 10.1186/ar2519 18822120 10.1186/ar2519 PMC2592803

[CR18] Hasimoglu YG, Chen X, Bakshi R, Schwarzschild MA, Macklin EA (2020) Does serum urate change as Parkinson’s disease progresses? J Parkinsons Dis 10(4):1571–1576. 10.3233/JPD-202064 32773396 10.3233/JPD-202064 PMC7683051

[CR19] Hoglinger GU, Respondek G, Stamelou M, Kurz C, Josephs KA, Lang AE, Mollenhauer B, Muller U, Nilsson C, Whitwell JL, Arzberger T, Englund E, Gelpi E, Giese A, Irwin DJ, Meissner WG, Pantelyat A, Rajput A, van Swieten JC, Troakes C, Antonini A, Bhatia KP, Bordelon Y, Compta Y, Corvol JC, Colosimo C, Dickson DW, Dodel R, Ferguson L, Grossman M, Kassubek J, Krismer F, Levin J, Lorenzl S, Morris HR, Nestor P, Oertel WH, Poewe W, Rabinovici G, Rowe JB, Schellenberg GD, Seppi K, van Eimeren T, Wenning GK, Boxer AL, Golbe LI, Litvan I, Movement Disorder Society-endorsed PSPSG (2017) Clinical diagnosis of progressive supranuclear palsy: the Movement Disorder Society criteria. Mov Disord 32(6):853–864. 10.1002/mds.26987 28467028 10.1002/mds.26987 PMC5516529

[CR20] Ikeda K, Nakamura Y, Kiyozuka T, Aoyagi J, Hirayama T, Nagata R, Ito H, Iwamoto K, Murata K, Yoshii Y, Kawabe K, Iwasaki Y (2011) Serological profiles of urate, paraoxonase-1, ferritin and lipid in Parkinson’s disease: changes linked to disease progression. Neurodegener Dis 8(4):252–258. 10.1159/000323265 21282940 10.1159/000323265

[CR21] Jack CR Jr., Andrews JS, Beach TG, Buracchio T, Dunn B, Graf A, Hansson O, Ho C, Jagust W, McDade E, Molinuevo JL, Okonkwo OC, Pani L, Rafii MS, Scheltens P, Siemers E, Snyder HM, Sperling R, Teunissen CE, Carrillo MC (2024) Revised criteria for diagnosis and staging of Alzheimer’s disease: Alzheimer’s Association Workgroup. Alzheimers Dement 20(8):5143–5169. 10.1002/alz.13859 38934362 10.1002/alz.13859 PMC11350039

[CR22] Jiang Z, Chen J, Wu S, Ji S, Yang Y, Fang W, Li Z, Lin J, Chen J, Wu C, Kwan HY, Lai Y, Zhao X (2024) Serum uric acid levels associated with outcomes of neurodegenerative disorders and brain health: findings from the UK Biobank. J Nutr Health Aging 28(9):100319. 10.1016/j.jnha.2024.100319 39094296 10.1016/j.jnha.2024.100319 PMC12880037

[CR23] Koros C, Simitsi AM, Papagiannakis N, Bougea A, Prentakis A, Papadimitriou D, Pachi I, Beratis I, Stanitsa E, Angelopoulou E, Antonelou R, Bregianni M, Lourentzos K, Papageorgiou SG, Bonakis A, Trapali XG, Stamelou M, Stefanis L (2023) Serum uric acid as a putative biomarker in prodromal Parkinson’s disease: longitudinal data from the PPMI study. J Parkinsons Dis 13(5):811–818. 10.3233/JPD-230007 37424476 10.3233/JPD-230007 PMC10473106

[CR24] Kovacs GG (2019) Molecular pathology of neurodegenerative diseases: principles and practice. J Clin Pathol 72(11):725–735. 10.1136/jclinpath-2019-205952 31395625 10.1136/jclinpath-2019-205952

[CR25] Kovacs GG, Milenkovic IJ, Preusser M, Budka H (2008) Nigral burden of alpha-synuclein correlates with striatal dopamine deficit. Mov Disord 23(11):1608–1612. 10.1002/mds.22207 18649394 10.1002/mds.22207

[CR26] Kovacs GG, Lukic MJ, Irwin DJ, Arzberger T, Respondek G, Lee EB, Coughlin D, Giese A, Grossman M, Kurz C, McMillan CT, Gelpi E, Compta Y, van Swieten JC, Laat LD, Troakes C, Al-Sarraj S, Robinson JL, Roeber S, Xie SX, Lee VM, Trojanowski JQ, Hoglinger GU (2020) Distribution patterns of tau pathology in progressive supranuclear palsy. Acta Neuropathol 140(2):99–119. 10.1007/s00401-020-02158-2 32383020 10.1007/s00401-020-02158-2 PMC7360645

[CR27] Lee Y, Park YH, Lee JJ, Sohn YH, Lee JM, Lee PH (2018) Gender-specific effect of uric acid on resting-state functional networks in de novo Parkinson’s disease. Parkinsonism Relat Disord 52:49–54. 10.1016/j.parkreldis.2018.03.023 29606606 10.1016/j.parkreldis.2018.03.023

[CR28] Lee YH, Chung SJ, Yoo HS, Lee Y, Sohn YH, Cha J, Lee PH (2020) Gender-specific effect of urate on white matter integrity in Parkinson’s disease. Parkinsonism Relat Disord 75:41–47. 10.1016/j.parkreldis.2020.05.012 32474403 10.1016/j.parkreldis.2020.05.012

[CR29] Litvan I, Campbell G, Mangone CA, Verny M, McKee A, Chaudhuri KR, Jellinger K, Pearce RK, D’Olhaberriague L (1997) Which clinical features differentiate progressive supranuclear palsy (Steele-Richardson-Olszewski syndrome) from related disorders? A clinicopathological study. Brain 120(Pt 1):65–74. 10.1093/brain/120.1.65 9055798 10.1093/brain/120.1.65

[CR30] Maetzler W, Stapf AK, Schulte C, Hauser AK, Lerche S, Wurster I, Schleicher E, Melms A, Berg D (2011) Serum and cerebrospinal fluid uric acid levels in Lewy body disorders: associations with disease occurrence and amyloid-beta pathway. J Alzheimers Dis 27(1):119–126. 10.3233/JAD-2011-110587 21765209 10.3233/JAD-2011-110587

[CR31] McFarland NR (2016) Diagnostic approach to atypical Parkinsonian Syndromes. Continuum Minneap Minn 22(4 Movement Disorders):1117–1142. 10.1212/CON.0000000000000348 27495201 10.1212/CON.0000000000000348 PMC5567217

[CR32] Merriman TR (2015) An update on the genetic architecture of hyperuricemia and gout. Arthritis Res Ther 17(1):98. 10.1186/s13075-015-0609-2 25889045 10.1186/s13075-015-0609-2 PMC4392805

[CR33] Moccia M, Pappata S, Erro R, Picillo M, Vitale C, Amboni M, Longo K, Palladino R, Barone P, Pellecchia MT (2015) Uric acid relates to dopamine transporter availability in Parkinson’s disease. Acta Neurol Scand 131(2):127–131. 10.1111/ane.12295 25288358 10.1111/ane.12295

[CR34] Oh M, Kim JS, Kim JY, Shin KH, Park SH, Kim HO, Moon DH, Oh SJ, Chung SJ, Lee CS (2012) Subregional patterns of preferential striatal dopamine transporter loss differ in Parkinson disease, progressive supranuclear palsy, and multiple-system atrophy. J Nucl Med 53(3):399–406. 10.2967/jnumed.111.095224 22323779 10.2967/jnumed.111.095224

[CR35] Oropesa-Ruiz JM, Huertas-Fernandez I, Jesus S, Caceres-Redondo MT, Vargas-Gonzalez L, Carrillo F, Carballo M, Gomez-Garre P, Mir P (2016) Low serum uric acid levels in progressive supranuclear palsy. Mov Disord 31(3):402–405. 10.1002/mds.26466 26686202 10.1002/mds.26466

[CR36] Sakuta H, Suzuki K, Miyamoto T, Miyamoto M, Numao A, Fujita H, Watanabe Y, Hirata K (2017) Serum uric acid levels in Parkinson’s disease and related disorders. Brain Behav 7(1):e00598. 10.1002/brb3.598 28127516 10.1002/brb3.598 PMC5256181

[CR37] Schirinzi T, Di Lazzaro G, Colona VL, Imbriani P, Alwardat M, Sancesario GM, Martorana A, Pisani A (2017) Assessment of serum uric acid as risk factor for tauopathies. J Neural Transm 124(9):1105–1108. 10.1007/s00702-017-1743-6 28620833 10.1007/s00702-017-1743-6

[CR38] Shen L, Ji HF (2013) Low uric acid levels in patients with Parkinson’s disease: evidence from meta-analysis. BMJ Open 3(11):e003620. 10.1136/bmjopen-2013-003620 24247326 10.1136/bmjopen-2013-003620 PMC3840343

[CR39] Squadrito GL, Cueto R, Splenser AE, Valavanidis A, Zhang H, Uppu RM, Pryor WA (2000) Reaction of uric acid with peroxynitrite and implications for the mechanism of neuroprotection by uric acid. Arch Biochem Biophys 376(2):333–337. 10.1006/abbi.2000.1721 10775420 10.1006/abbi.2000.1721

[CR40] van Wamelen DJ, Taddei RN, Calvano A, Titova N, Leta V, Shtuchniy I, Jenner P, Martinez-Martin P, Katunina E, Chaudhuri KR (2020) Serum uric acid levels and non-motor symptoms in Parkinson’s disease. J Parkinsons Dis 10(3):1003–1010. 10.3233/JPD-201988 32444561 10.3233/JPD-201988

[CR41] Wang M, Tang Z (2024) No causal relationship between serum urate and neurodegenerative diseases: a Mendelian randomization study. Exp Gerontol 194:112503. 10.1016/j.exger.2024.112503 38955238 10.1016/j.exger.2024.112503

[CR42] Weisskopf MG, O’Reilly E, Chen H, Schwarzschild MA, Ascherio A (2007) Plasma urate and risk of Parkinson’s disease. Am J Epidemiol 166(5):561–567. 10.1093/aje/kwm127 17584757 10.1093/aje/kwm127 PMC2391073

[CR43] Wen M, Zhou B, Chen YH, Ma ZL, Gou Y, Zhang CL, Yu WF, Jiao L (2017) Serum uric acid levels in patients with Parkinson’s disease: a meta-analysis. PLoS ONE 12(3):e0173731. 10.1371/journal.pone.0173731 28319195 10.1371/journal.pone.0173731 PMC5358777

[CR44] Zhang J, Zeng L, Bu L, Liao H, Wang M, Xiong Y, Cao F (2024a) Association between high uric acid and the risk of Parkinson’s disease: a meta-analysis. Med (Baltim) 103(30):e38947. 10.1097/MD.0000000000038947 10.1097/MD.0000000000038947 PMC11272381 39058857

[CR45] Zhang T, An Y, Shen Z, Yang H, Jiang J, Chen L, Lu Y, Xia Y (2024b) Serum urate levels and neurodegenerative outcomes: a prospective cohort study and Mendelian randomization analysis of the UK Biobank. Alzheimers Res Ther 16(1):106. 10.1186/s13195-024-01476-x 38730474 10.1186/s13195-024-01476-x PMC11088014

