# Supplementary figures and images for: Longitudinal serum uric acid levels are not associated with dopamine transporter binding in progressive supranuclear palsy
Source: J Neural Transm (Vienna). 2026 Apr 6;133(7):1543–51. doi: 10.1007/s00702-026-03141-z (PMC13428700; doi:10.1007/s00702-026-03141-z)

## Slide 1
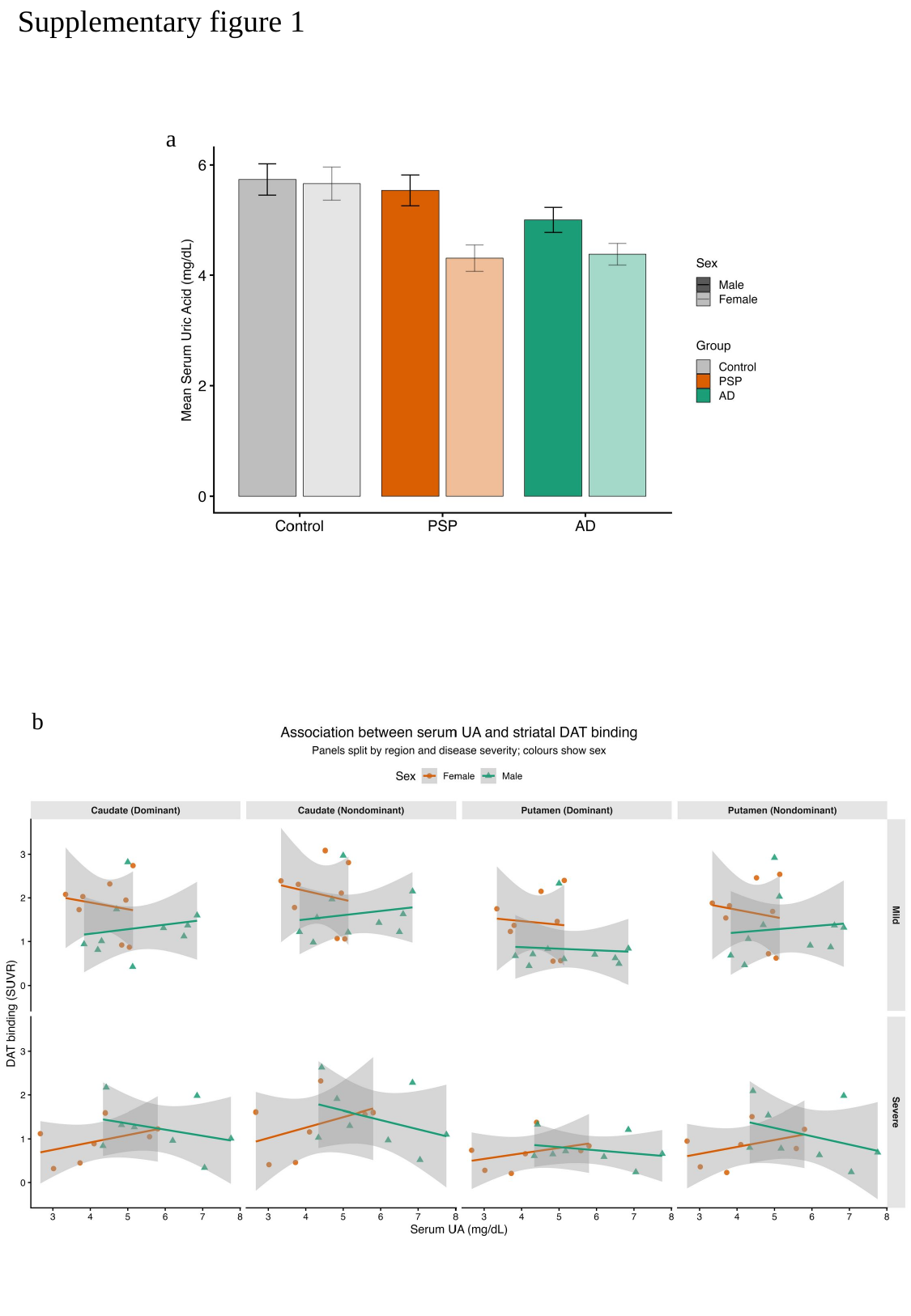

Supplementary figure 1
a
b

Supplement: Supplementary file 1 — Supplementary Material 1 Supplementary Fig. 1. (a) Mean serum UA levels stratified by sex and diagnostic group. Bars represent group means, and error bars indicate standard error of the mean (SEM). Darker bars represent males, lighter bars represent females. (b) Associations between serum uric acid and striatal dopamine transporter binding in PSP patients stratified by disease severity and sex. Scatter plots showing the relationship between serum UA levels (x-axis) and striatal DAT binding ratios (y-axis, measured as standardized uptake value ratio, SUVR) across four brain regions. Data are stratified by disease severity (Mild: top row; Severe: bottom row) and sex (Female: orange circles; Male: green triangles). Each panel represents a different striatal region: Caudate (more_affected), Caudate (less_affected), Putamen (more_affected), and Putamen (less_affected). Linear regression lines with 95% confidence intervals (shaded areas) are shown for each sex. In mild disease, female patients generally showed positive associations between UA and DAT binding (particularly in caudate regions), while male patients showed relatively flat or negative trends. In severe disease, both sexes showed predominantly flat or negative associations across all regions, suggesting that the neuroprotective association of UA may be disease stage-dependent. None of the associations reached statistical significance [file 702_2026_3141_MOESM1_ESM.pptx]
