# Supplementary material for: Longitudinal serum uric acid levels are not associated with dopamine transporter binding in progressive supranuclear palsy
Source: J Neural Transm (Vienna). 2026 Apr 6;133(7):1543–51. doi: 10.1007/s00702-026-03141-z (PMC13428700; doi:10.1007/s00702-026-03141-z)
Supplement: Supplementary file 2 — Supplementary Material 2 [file 702_2026_3141_MOESM2_ESM.docx]

**Supplementary Table 1.** Posterior estimates from Bayesian measurement-error–adjusted regression models examining associations between uric acid (UA) intercept and slope and regional striatal DAT binding (caudate/putamen; more_affected/less_affected). Posterior means with corresponding estimation error and 95% credible intervals (CI). Abreviations: UA, uric acid; DAT, dopamine transporter; CI, credible interval; me, measurement error.

| **Model** | **Parameter** | **Mean** | **Est_Error** | **CI_low** | **CI_high** |
| --- | --- | --- | --- | --- | --- |
| Caudate (less_affected) | UA intercept (me) | 0.044342 | 0.07519 | -0.10302 | 0.1910 |
| Caudate (less_affected) | UA slope (me) | -0.049546 | 1.99053 | -3.99191 | 3.8175 |
| Caudate (more_affected) | UA intercept (me) | 0.065754 | 0.07382 | -0.07811 | 0.2099 |
| Caudate (more_affected) | UA slope (me) | 0.006859 | 1.97954 | -3.95939 | 3.8275 |
| Putamen (more_affected) | UA intercept (me) | 0.055818 | 0.07254 | -0.08450 | 0.2013 |
| Putamen (more_affected) | UA slope (me) | -0.068662 | 1.97557 | -3.97201 | 3.8591 |
| Putamen (less_affected) | UA intercept (me) | 0.091729 | 0.07541 | -0.05308 | 0.2380 |
| Putamen (less_affected) | UA slope (me) | -0.073638 | 2.01014 | -3.98567 | 3.9000 |
